# Performance optimization and mechanism study of asphalt mixtures modified with ZM additive

**DOI:** 10.1371/journal.pone.0337535

**Published:** 2025-12-01

**Authors:** Yining Wang, Weishuai Ji

**Affiliations:** 1 School of Transportation Science and Engineering, Harbin Institute of Technology, Harbin, China; 2 Highway Construction Center of Heilongjiang Province, Harbin, China; 3 School of Civil Engineering and Transportation, Northeast Forestry University, Harbin, China; Shandong University of Technology, CHINA

## Abstract

To enhance the high-temperature stability, low-temperature crack resistance, moisture susceptibility, and fatigue life of asphalt mixtures, this study systematically investigates the effects of Zhongmao Modifier (ZM), a solid granular direct-to-asphalt polymer–resin additive produced by Zhongmao Company (Shenyang, China), dosage and mixing process parameters on the pavement performance of asphalt mixtures and elucidates the underlying mechanisms. Orthogonal experiments determined the optimal mixing parameters as a mixing temperature of 170°C, dry mixing time of 180 s, and wet mixing time of 240 s. Experimental results show that the ZM modifier significantly improves the dynamic stability and rutting resistance of the mixture, with dynamic stability increasing to 5245 and rut depth decreasing to 2.26 mm at a dosage of 0.5%. The low-temperature flexural strain increases while the bending stiffness decreases, indicating improved crack resistance. In terms of moisture stability, both the retained stability and freeze–thaw splitting strength ratio outperform those of the base asphalt, reaching 88% and 90%, respectively. Fatigue test results reveal that the ZM modifier markedly extends fatigue life, with a maximum increase of 128.9%, and reduces fatigue sensitivity to the stress ratio. Displacement evolution analysis indicates that the modifier enhances inter-aggregate bonding, increases failure displacement, prolongs the stable phase, and significantly improves overall durability. Mechanistic analysis suggests that the polymer–resin components of the ZM modifier form a stable elastic network within the asphalt matrix and strengthen the interfacial bonding between asphalt and aggregates, thereby improving high-temperature stability and durability while maintaining low-temperature flexibility. The findings provide theoretical support and practical guidance for the broader application of ZM-modified asphalt mixtures in real-world pavement engineering, particularly for enhancing the performance and service life of road surfaces under varying environmental conditions.

## 1. Introduction

The occurrence of distresses in asphalt pavements has drawn increasing attention from researchers. It has been discovered that the performance of asphalt and asphalt mixtures can be improved by incorporating certain additives into the asphalt, which has led to the development and application of asphalt modifiers. Over time, a wide variety of modifiers have been developed for use in asphalt pavements. In recent years, some scholars have begun to explore the direct incorporation of modifiers into asphalt mixtures during mixing—a process referred to as using externally added modifiers or direct-to-mix modifiers. Researchers have studied the effects of different types and dosages of such modifiers on the performance of both asphalt and asphalt mixtures, analyzing their modification mechanisms and the associated changes in process parameters. These efforts aim to optimize the application of direct-to-mix modifiers in asphalt pavement engineering.

Asphalt pavements often suffer from rutting at high temperatures, cracking at low temperatures, and moisture-induced damage under freeze–thaw cycles, which significantly shorten service life and increase maintenance costs [1]. To mitigate these distresses, polymer-based modifiers such as styrene–butadiene–styrene (SBS), crumb rubber, and composite resins have been widely applied [2,3]. However, SBS-modified asphalt shows limitations in storage stability and low-temperature performance, while crumb rubber suffers from compatibility and workability issues [4]. Composite additives may enhance specific properties but often face high costs and uncertain long-term durability [5]. Therefore, it is necessary to develop new types of modifiers that can simultaneously improve high-temperature rutting resistance, low-temperature cracking resistance, and moisture stability of asphalt mixtures.

Beginning in the mid-20th century, international scholars conducted extensive experimental studies on asphalt modifiers. Initial efforts focused on enhancing the fundamental properties of asphalt to produce asphalt mixtures with superior performance. Among the various types of modifiers, polymer-based modifiers—such as resins and synthetic polymers—have received the most attention due to their effectiveness and widespread use [6]. However, the wet pre-mixing modification process has certain technical limitations, including issues such as modifier-asphalt segregation, decline in anti-aging performance, and difficulties in quality control during production and regulation [7,8]. With the continuous development of new modifier materials, researchers have sought to identify and develop modifiers that can be directly added into the asphalt mixing plant during the production of asphalt mixtures, offering a more efficient and stable modification approach.

The research and application of direct-to-mix modifier technology originated in Europe in the 20th century, with France taking the lead in pioneering efforts. France developed relevant technologies and established official production and construction standards for such materials [9]. Since then, countries including the United States, the United Kingdom, and Finland have successively launched studies on direct-to-mix modifier technologies beginning in the 20th century [10].

Research dating back to the 1960s indicated that asphalt mixtures produced using pre-modified binders generally exhibit superior performance compared to those prepared by directly adding modifiers to the mix [11]. However, it wasn’t until the late 20th century that the United States achieved a significant breakthrough in the field of direct-to-mix modifiers, marking a shift in research focus. This led to direct-to-mix modifiers becoming a prominent research topic.

During the same period, many European countries also began developing direct-to-mix modifiers. Notable examples include the PR-Module from France, Lufu 8000 and DUROFLEX from Germany, and sulfur-based modifiers developed by Shell. These modifiers effectively enhanced the high-temperature performance of asphalt mixtures without requiring additional equipment. They also offered advantages in storage, transport, and economic efficiency, making them more practical than traditional asphalt modification methods.

With the continuous development of direct-to-mix technologies, several researchers have advanced the field: Shu Yi [12] investigated the functional mechanisms of direct-to-mix modifiers in asphalt mixture production and identified key quality control parameters, offering practical guidance for field applications. Zhang et al. [13] studied the performance of dry-process modifiers and determined the optimal mixing process through experimental analysis.

Shanghai Highway & Bridge Co., Ltd. [14] analyzed the influence of high-viscosity direct-to-mix modifiers on pavement performance and validated the modification effectiveness using the Kentaburg scattering test. Zhang [15] examined the impact of a novel TPS modifier on asphalt, conducting high- and low-temperature performance tests on asphalt modified with different TPS dosages. The study confirmed the effectiveness of the modifier and recommended a dosage range of 13% to 18%. Xu Haonan [16] studied the preparation of LM-S direct-to-mix modified asphalt and used FTIR analysis to show that LM-S does not generate new functional groups but increases the quantity of existing ones. This increases enhanced asphalt polarity and improved its adhesive strength.

With the continuous increase in traffic load, the durability of pavements using direct-to-mix modified asphalt mixtures has become a central concern among researchers. Xu Kai [17] conducted semi-circular bending (SCB) fatigue tests under five different stress levels and analyzed the relationship between mechanical strength and fatigue performance by establishing fatigue equations. Wang [18] investigated the effects of loading frequency and temperature on the fatigue life of asphalt mixtures. The results showed that higher loading frequencies led to longer fatigue life, whereas elevated temperatures accelerated crack propagation and reduced fatigue resistance. Li Youyun [19] developed a finite element model for SCB specimens and employed a multiscale iterative approach to predict the flexural tensile strength at various levels of asphalt mixture. The study found that during fatigue damage, the flexural strength decays slowly in the early stages but deteriorates rapidly in the later stages.

Jiang [20] simulated water–temperature cycling using freeze–thaw cycles to evaluate the durability of hot-recycled asphalt mixtures with varying reclaimed material contents. The results indicated that recycled mixtures exhibit inferior durability under long-term water–temperature cycling compared to virgin mixtures, and higher reclaimed content further reduces durability. Zhang [21] performed both SCB and Indirect Tensile Fatigue (IDT) tests to evaluate the fatigue performance of fiber-modified asphalt mixtures. By analyzing the displacement curves of the specimens during fatigue testing, it was found that the addition of fibers significantly enhances fatigue life.

The mechanical performance of asphalt mixtures is influenced by various factors such as load magnitude, loading frequency, loading duration, and temperature. Many researchers have investigated the viscoelastic behavior of asphalt mixtures through experimental studies and mathematical modeling.

Kuchiishi [22] conducted dynamic modulus tests on cold recycled asphalt mixtures and constructed master curves to analyze their viscoelastic properties. The study demonstrated that the temperature-dependent stiffness of cold recycled mixtures can significantly affect pavement mechanical performance.

Yasmina [23] evaluated the dynamic mechanical properties of asphalt mixtures produced with crumb rubber via the dry process. The results showed that under high-frequency and low-temperature conditions, mixtures with rubber exhibited relatively low dynamic modulus values.

Kuna [24] performed dynamic modulus tests under various temperatures, frequencies, and stress levels. Based on the time–temperature superposition principle, they developed dynamic modulus master curves, which effectively captured the viscoelastic response of asphalt mixtures and enabled accurate prediction of dynamic modulus values.

Kang [25] investigated the variation patterns of dynamic modulus curves for three types of crumb rubber/SBS composite modified asphalt mixtures. The results indicated that the inclusion of crumb rubber enhances the dynamic modulus of the mixture, and that the modulus increases proportionally with the rubber content.

Recent developments in polymer–resin–based asphalt modifiers have demonstrated significant potential for improving the mechanical and durability properties of asphalt mixtures. Studies on polymer–resin systems, nanocomposite modifiers, and reactive polymers have shown enhanced network structures, better interfacial adhesion, and improved aging resistance [add several references, e.g., Construction and Building Materials, 2022–2024]. These advancements provide a relevant context for understanding the multifunctional performance of the Zhongmao (ZM) modifier examined in this study.

In this context, the Zhongmao (ZM) additive investigated in this study is a novel polymer–resin–based direct-to-mix modifier, which aims to overcome the limitations of traditional modifiers and improve the overall durability of asphalt mixtures. The present work systematically evaluates its influence on high-temperature rutting resistance, low-temperature cracking resistance, moisture stability, and fatigue life, while also exploring the underlying mechanism of performance enhancement.

## 2. Experimental materials and methods

### 2.1. Experimental materials

(1) Asphalt

The asphalt used in this study is 90# base asphalt produced in Panjin, Liaoning Province. Its fundamental performance indicators are listed in Table 1.

**Table 1:** Basic performance indicators of base asphalt.

Test Item	Measured Value	Specification Requirement
Penetration at 25°C (0.1 mm)	82.8	80-100
Ductility at 15°C (cm)	106	≥100
Softening Point (°C)	47.5	≥44
Dynamic Viscosity at 60°C (Pa·s)	230	≥160
Flash point (°C)	260	≥245

(2) Coarse and fine aggregates

The aggregates used in this study are basalt. In asphalt mixtures, coarse aggregates serve as the structural skeleton, and their strength directly determines the overall strength of the mixture. Fine aggregates function to absorb asphalt, fill the voids between coarse particles, and improve the mixture’s compactness. The basic properties of the aggregates are listed in Table 2 and Table 3.

**Table 2:** Technical indicators of coarse aggregate.

Test Item		Unit	Tested Value	Specification Requirement	Test Method
Apparent Relative Density	16 mm	g/cm^3^	2.798	≥2.6	T0304
	13.2 mm		2.796		T0304
	9.5 mm		2.786		T0304
	4.75 mm		2.771		T0304
	2.36 mm		2.76		T0304
Water absorption rate	16 mm	%	0.272	≤2	T0304
	13.2 mm	%	0.288		T0304
	9.5 mm	%	0.328		T0304
	4.75 mm	%	0.567		T0304
	2.36 mm	%	0.652		T0304
Crushing Value of Aggregate		%	10.2	≤26	T0316
Los Angeles Abrasion Value		%	12.5	≤28	T0317
Flat and Elongated Particle Content > 9.5 mm		%	6.7	≤12	T0312
Flat and Elongated Particle Content≤9.5 mm		%	9.4	≤18	T0312
Fines Content < 0.075 mm		%	0.29	≤1	T0310

**Table 3:** Technical specifications of fine aggregates.

Test item		Unit	Measured value	Specification requirement	Test Method
Apparent relative density	2.36 mm	g/cm^3^	2.751	≥2.5	T0328
	1.18 mm		2.753		T0328
	0.6 mm		2.746		T0328
	0.3 mm		2.741		T0328
	0.15 mm		2.738		T0328
	0.075 mm		2.736		T0328
Clay content (<0.075 mm)		%	0.71	≤3	T0333
Soundness (portion >0.3 mm)		%	8.5	≤15	T0340
Angularity (flow time)		s	46	≥30	T0345

(3) Mineral filler

Mineral filler plays a dual role in asphalt mixtures by filling voids and interacting with asphalt to form a mastic. In this study, limestone powder was used as the mineral filler. Its basic properties are listed in Table 4.

**Table 4:** Technical specifications of mineral filler.

Test item		Unit	Measured value	Specification requirement	Test Method
Apparent density		g/cm^3^	2.686	≥2.5	T0352
Hydrophilic coefficient		—	0.83	≤1	T0353
Moisture content		%	0.19	≤1	T0103
Appearance		—	No agglomeration	No agglomeration	—
Particle Size	<0.6mm	%	100	100	T0351
	<0.15mm	%	95.3	90 ~ 100	
	<0.075mm	%	90.3	75 ~ 100

(4) Modifier

**Fig 1:**
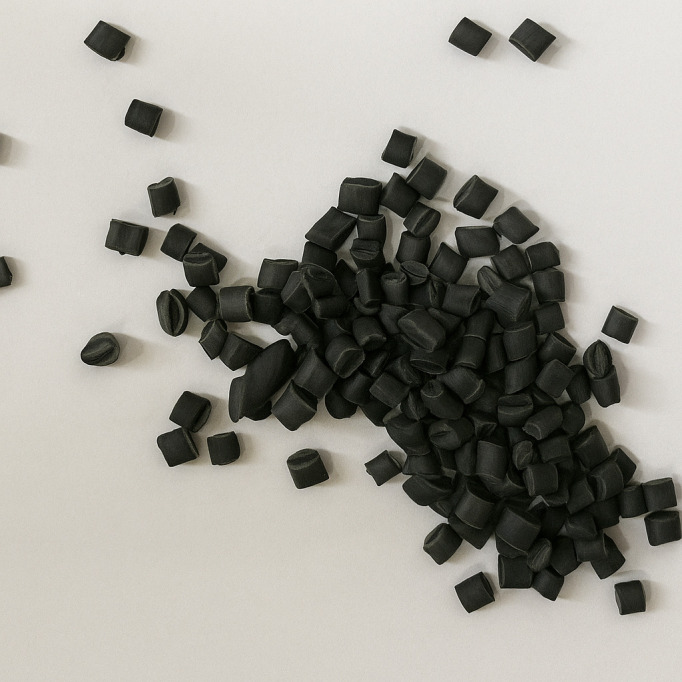
ZM Modifier.

The modifier investigated in this study is the ZM modifier, a solid granular direct-to-mix high-polymer additive specifically designed for asphalt mixtures. It is primarily composed of high-performance polymers and resins and is produced in Shenyang, Liaoning Province. Owing to its ability to significantly enhance pavement performance and simplify construction procedures, the ZM modifier has been widely applied in road engineering projects across various regions in China in recent years, yielding favorable results. The ZM modifier appears as black granules, as shown in Fig 1. Its technical specifications are provided in Table 5.

**Table 5:** Technical Specifications of ZM Modifier.

Item	Technical Requirement
Appearance	Black solid granules
Main Components	45%polymer, 50%resin, 5% reactants
Particle Size	<5.8mm
Softening Point	137°C
Density	0.95g/cm3

### 2.2. Research methods

#### 2.2.1. Mix design.

(1) Gradation Curve

As the primary material used in highway pavements, asphalt mixtures contain a high volume fraction of aggregate, which also bears the majority of external loads. Therefore, aggregate plays a crucial role in determining the overall performance of asphalt mixtures. A well-designed aggregate gradation can significantly enhance the road performance of the mixture.

In this study, an S-shaped gradation curve was adopted for the mix design. This type of gradation improves the mixture’s high-temperature stability while ensuring it meets other functional requirements for pavement use. During preparation, the amount of coarse aggregate near the nominal maximum particle size was reduced, and the content of fine particles smaller than 0.6 mm was limited. The proportion of mineral filler was also carefully controlled. Increasing the proportion of medium-sized particles in the 10–15 mm range helps form an S-shaped gradation curve, which contributes to greater surface texture depth and improved rutting resistance at high temperatures. Research has shown that a gently sloping S-type gradation curve offers excellent high-temperature stability to the mixture.

**Fig 2:**
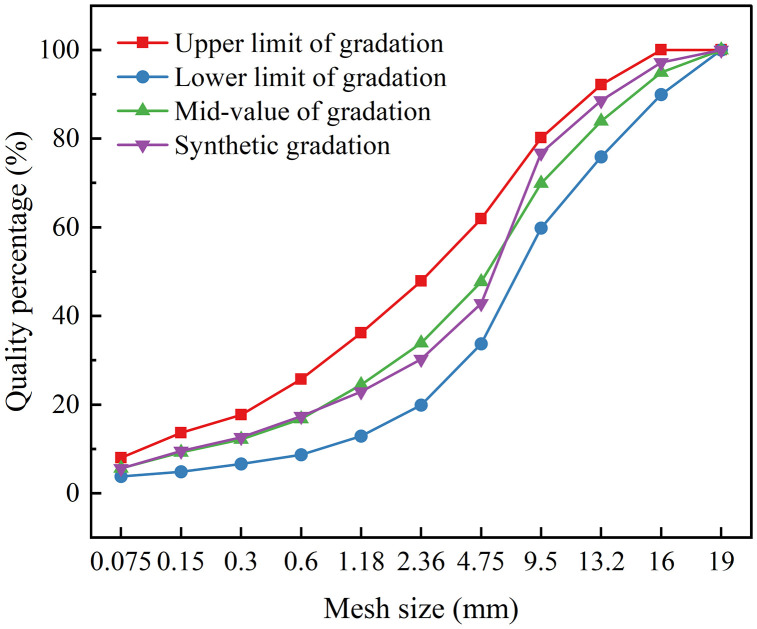
Gradation curve of asphalt mixture.

According to relevant standards, the selected gradation type for this study is AC-16. The aggregate was proportioned by weighing and recombining individual size fractions to meet the target gradation. The gradation data for the asphalt mixture are provided in Table 6, and the corresponding gradation curve is shown in Fig 2.

**Table 6:** Gradation composition of asphalt mixture.

Sieve size/mm	16	13.2	9.5	4.75	2.36	1.18	0.6	0.3	0.15	0.075
Upper limit (%)	100	92	80	62	48	36	26	18	14	8
Lower limit (%)	90	76	60	34	20	13	9	7	5	4
Target gradation (%)	97.1	88.6	76.9	42.9	30.6	23.1	17.7	12.7	9.7	6.2

(2) Determination of optimum asphalt content

**Fig 3:**
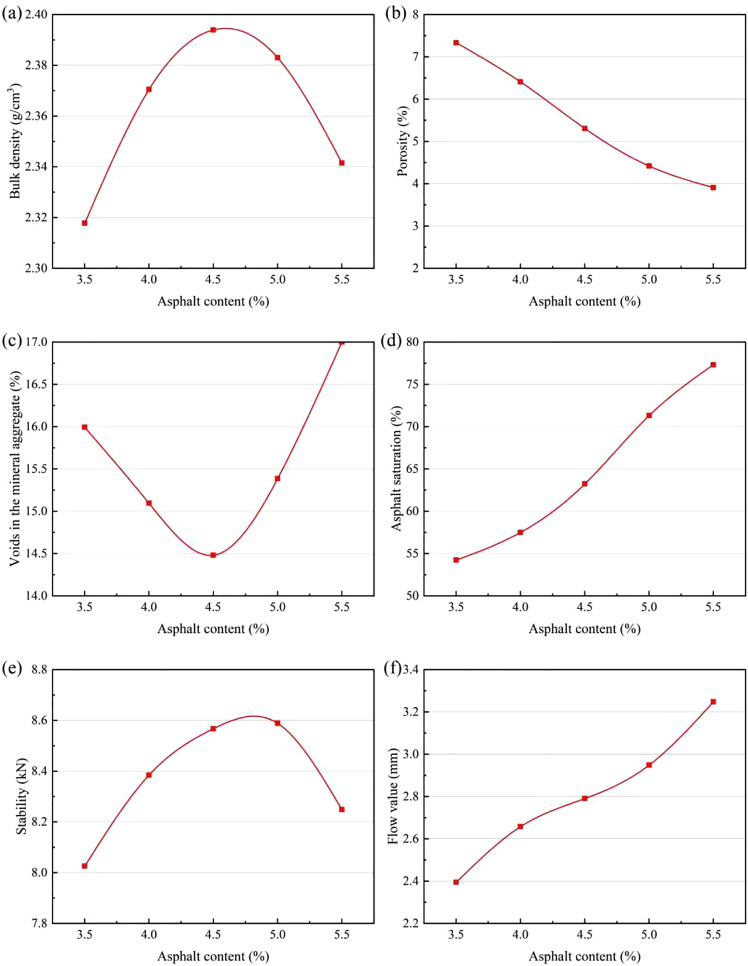
Gradation curve of asphalt mixture Relationship between asphalt content and pavement performance characteristics: (a) Bulk density, (b) Air voids, (c) Voids in mineral aggregate, (d) Asphalt saturation, (e) Stability, (f) Asphalt Content.

Asphalt content significantly affects pavement performance. Excessive asphalt can lead to problems such as bleeding, while insufficient asphalt often results in inadequate strength and poor moisture stability. Therefore, the optimum asphalt content must be determined through the Marshall test. In this section, the optimal asphalt–aggregate ratio for dense-graded asphalt mixtures was determined using laboratory Marshall tests. A median asphalt content of 4.5% was selected, and five asphalt contents were tested at 0.5% intervals: 3.5%, 4.0%, 4.5%, 5.0%, and 5.5%. Marshall specimens were prepared accordingly, demolded after 12 hours, and checked for compliance with the standard height requirement (63.5 ± 1.3 mm). Following standard procedures, technical indices such as bulk density were measured. The results of the Marshall tests are provided in Table 7, and the test data were plotted with asphalt content on the horizontal axis and each corresponding index on the vertical axis to generate performance curves, as shown in Fig 3.

**Table 7:** Gradation composition of asphalt mixture.

Asphalt content(%)	Bulk density(g/cm^3^)	Air voids (%)	Void ratio (%)	Asphalt saturation (%)	Stability(kN)	Flow (0.1 mm)
3.5	2.328	7.3	16	54.3	8.03	23.9
4	2.365	6.4	15.1	57.7	8.39	26.5
4.5	2.394	5.3	14.5	63.5	8.51	27.8
5	2.383	4.4	15.4	71.4	8.59	29.4
5.5	2.342	3.9	17.3	77.4	8.25	32.3

From the graph, the asphalt content corresponding to the maximum bulk density is a_1_ = 4.7, and the asphalt content corresponding to the maximum stability is a_2_ = 4.8. The asphalt content associated with the median target air voids is a_3_ = 4.8, while that corresponding to the median asphalt saturation is a_4_ = 4.9. Taking the average of these four values yields OAC_1_ = 4.8.

According to the Marshall technical specifications for asphalt mixtures, OAC_min_ = 4.6, OAC_max_ = 5.3. The median of this range is OAC_2_ = 4.95. Finally, the optimal asphalt content for the base asphalt is determined by OAC=(OAC_1_ + OAC_2_)/2=(4.8% + 4.95%)/2 = 4.87%

#### 2.2.2. Mineral filler characteristics.

(3) Preparation of modified asphalt

**Fig 4:**
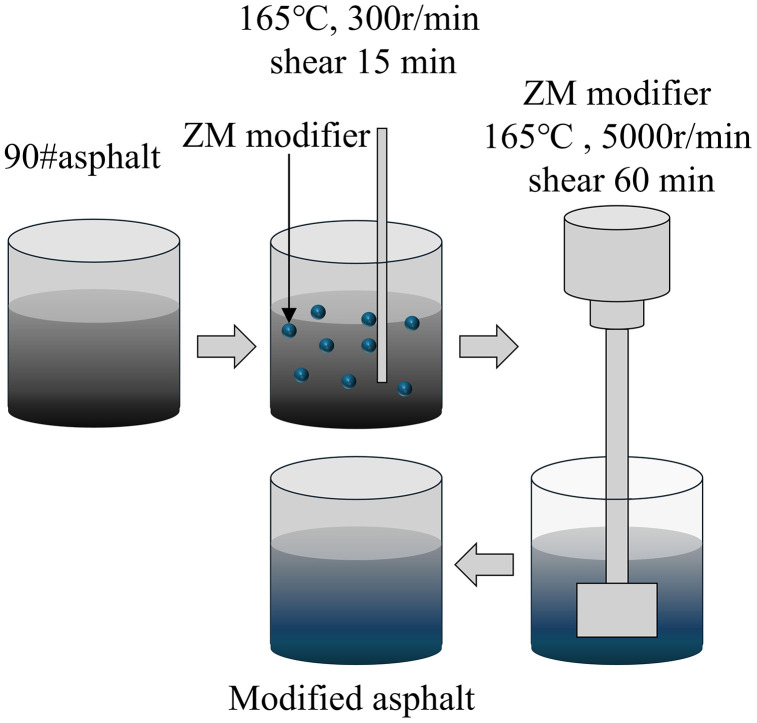
Preparation flowchart of ZM-modified asphalt.

In this section, ZM-modified asphalt was prepared by directly incorporating the ZM modifier into base asphalt at dosage rates of 3%, 5%, and 7% by weight of the asphalt. The preparation process combined mechanical stirring and high-speed shearing. The specific procedure is outlined below, and the preparation flowchart is shown in Fig 4:

(a) The base asphalt was heated until molten and poured into a metal container.
(b) The ZM modifier was added in the designated amounts. The container was placed on a 165°C electric hotplate, and the asphalt stirrer was activated. The stirring speed was gradually increased to 300 r/min, and the mixture was stirred at this speed for 15 minutes.
(c) The pre-mixed asphalt was then subjected to high-temperature shearing at 160°C using a high-speed shear mixer operating at 5000 r/min for 60 minutes.

#### 2.2.2. Test methods.

To ensure experimental reliability, all tests were conducted with three parallel specimens for each condition, and the average value was reported. When significant deviations were observed, additional specimens were tested. The variability in results is represented as mean ± standard deviation. Statistical significance of the differences between the base asphalt and ZM-modified asphalt mixtures was evaluated using one-way analysis of variance with a 95% confidence level.

(1) Design of Orthogonal Experiment

Orthogonal experimental design is a method used to study the effects of multiple factors at multiple levels. By selecting representative combinations of data points, it allows researchers to efficiently and economically explore the influence of different variables while reducing the number of experimental trials. This method helps identify optimal parameter settings with high accuracy [26].

In this study, an orthogonal experiment was designed to investigate three key factors that significantly affect the performance of ZM-modified asphalt mixtures: T, mixing temperature; t₁, dry mixing time between ZM modifier and aggregates; and t₂, mixing time after the addition of asphalt and mineral filler. Due to the high variability in experimental results, all procedures were carried out by the same operator to minimize inconsistency. Unless otherwise specified, the dosage of the ZM modifier in this study is expressed as a percentage by total mass of aggregates and mineral filler. Accordingly, the experiments were conducted using the ZM modifier at a dosage of 0.3% by mass of aggregates and mineral filler. The selected levels for each factor were as follows: Mixing temperature: 160°C, 170°C, and 180°C; dry mixing time (t₁): 120 s, 180 s, and 240 s; and wet mixing time (t₂): 120 s, 180 s, and 240 s. The factor–level combinations are shown in Table 8. The orthogonal experiment, conducted at a ZM dosage of 0.3% by mass of aggregates and mineral filler, yielded the optimal mixing parameters of 170°C, 180 s, and 240 s, respectively.

**Table 8:** Factor-level.

Level	*T*(°C)	*t*_1_(s)	*t*_2_(s)
1	160	240	120
2	170	180	180
3	180	120	240

It should be noted that the addition of ZM modifier did not affect the determination procedure of the optimum asphalt content, which was obtained following the standard Marshall design method. Within the studied dosage range, the ZM modifier had no significant influence on the optimum asphalt content.

The experiment used dynamic stability as the control index, which is a key parameter reflecting the rutting resistance and overall road performance of asphalt mixtures—a higher value indicates better performance. Three primary factors were investigated: mixing temperature, dry mixing time of the modifier, and total mixing time after the addition of asphalt and mineral filler. By applying the principles of orthogonal experimental design and using the orthogonal table Lg(3³), the number of experimental trials was significantly reduced while ensuring a systematic and balanced arrangement of test conditions, shown in Table 9. This approach enabled efficient identification of optimal parameter combinations and yielded reliable experimental outcomes.

**Table 9:** Orthogonal experimental design.

Test number	*T*(°C)	*t*_1_(s)	*t*_*2*_(s)
1	1 (160)	1 (240)	1 (120)
2	1	2 (180)	2 (180)
3	1	3 (120)	3(240)
4	2 (170)	2	3
5	2	3	1
6	2	1	2
7	3 (180)	3	2
8	3	1	3
9	3	2	1

(2) High-Temperature Stability Test

**Fig 5:**
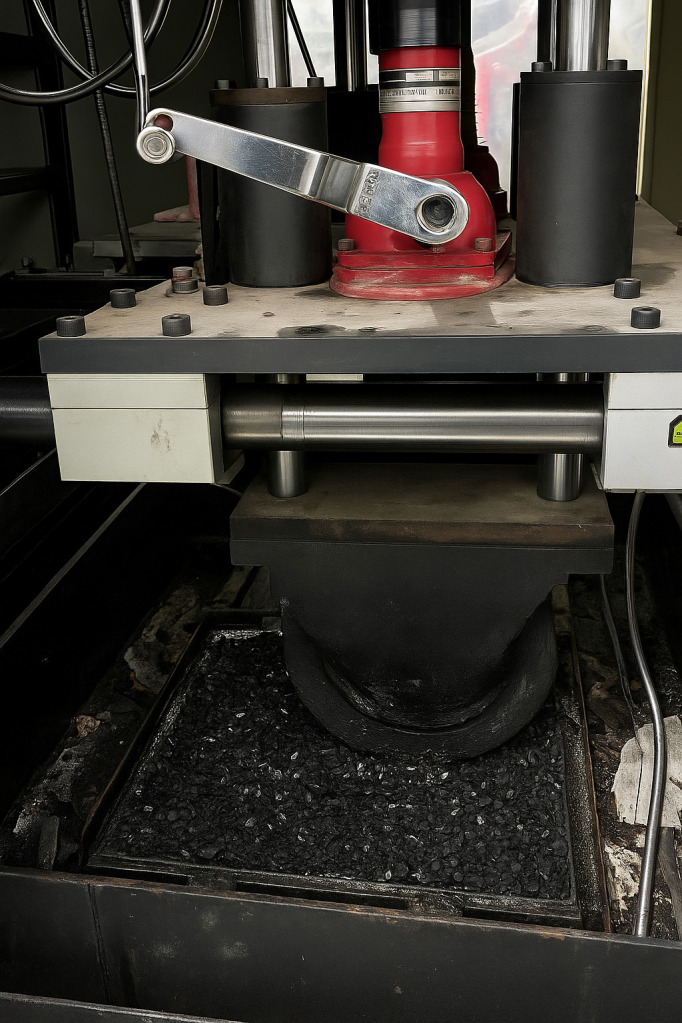
Rutting test machine.

**Fig 6:**
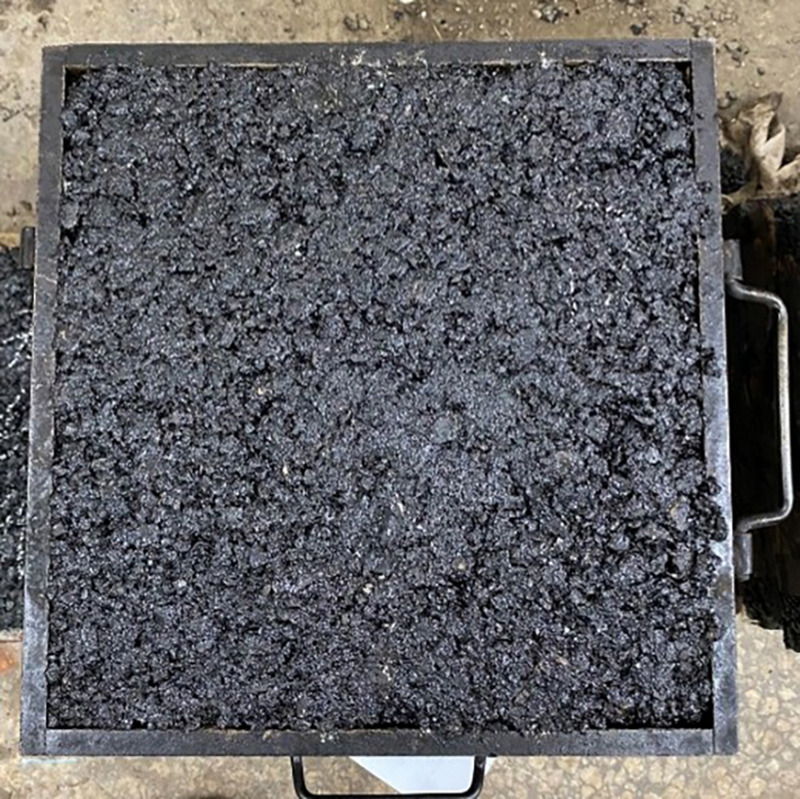
Rutting specimen.


This study employed the rutting test to evaluate the performance of asphalt mixtures. The rutting test can realistically simulate the deformation of actual pavement under the combined effects of vehicular loading and thermal stress, thus better representing the real rutting resistance of asphalt pavement. As shown in Fig 5, the experimental device used for the tests is described in detail, and the specimens are presented in Fig 6. The high-temperature performance of the asphalt mixture was assessed based on two parameters obtained from the test: dynamic stability and rut depth. The wheel-tracking method was used to prepare asphalt mixture rutting slabs with dimensions of 300 mm × 300 mm × 50 mm. Prior to testing, the specimens were conditioned at a constant temperature of 60°C ± 1°C for 5 hours to ensure uniform internal and external temperatures. During the rutting test, a wheel-tracking device was used to apply loading, maintaining a test temperature of 60°C ± 1°C, a wheel pressure of 0.7 ± 0.05 MPa, and a loading speed of 42 passes per minute. The test instrument automatically recorded the rut depth–time curve. The rut depths at 45 minutes (t₁) and 60 minutes (t₂), denoted as d₁ and d₂ respectively, were used to calculate the dynamic stability of the asphalt mixture, according to Equation (1).

$$DS=\frac{\left(t_{2}-t_{1}\right)\times N}{d_{2}-d_{1}}\times c_{1}\times c_{2}$$ (1)

In the equation:

*c*_*1*_—Specimen machine type coefficient, taken as 1.0;

*c*_*2*_—Specimen coefficient, taken as 1.0;

*N*—Wheel reciprocating compaction rate during the test, taken as 42 passes/min.

(3) Low-temperature cracking performance test

**Fig 7:**
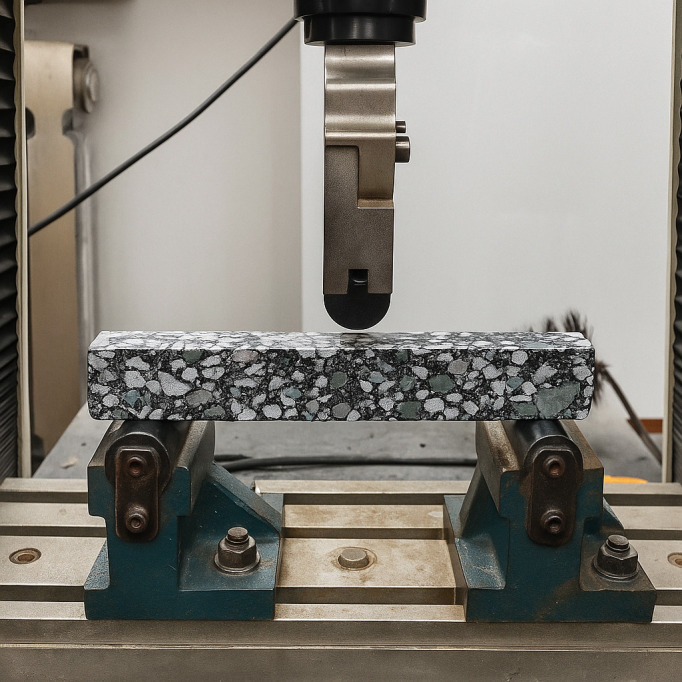
Small beam bending specimen and testing equipment.

Small beam specimens (30 mm × 35 mm × 250 mm) were prepared using base asphalt, SBS-modified asphalt, and ZM-modified asphalt mixtures, as shown in Fig 7. Prior to testing, the specimens were conditioned at the specified temperature for 4 hours. A universal testing machine was employed to perform a low-temperature bending test on the asphalt mixtures, aiming to assess their cracking resistance under low-temperature conditions. A beam mold with a span of 200 mm was used, and the loading rate was set to 50 mm/min at a constant temperature of −10°C. Each test condition was repeated three times to ensure consistent results.

The flexural strength, failure strain, and stiffness modulus at fracture were calculated using the following formulas. The average values from the three tests were used to evaluate the performance of the mixtures under low-temperature conditions.


$$R_{B}=\frac{3\times L\times P_{B}}{2\times b\times h^{2}}$$ (2)

$$\varepsilon_{B}=\frac{6\times L\times d}{L^{2}}$$ (3)

$$S_{B}=\frac{R_{B}}{\varepsilon_{B}}$$ (4)

In the equation:

*R*_*B*_—Flexural tensile strength at failure of the specimen, in MPa

*ε*_*B*_—Maximum tensile strain at failure of the specimen, in με

*S*_*B*_—Flexural stiffness modulus at failure of the specimen, in MPa

*P*_*B*_—Maximum load at failure of the specimen, in N

*b*—Width of the specimen at the fracture section, in mm

*h*—Height of the specimen at the fracture section, in mm

*L*—Span length of the specimen, taken as 200 mm

*d*—Mid-span deflection of the specimen at failure, in mm

(4) Moisture Stability Test
  (a) Immersion Marshall Test

**Fig 8:**
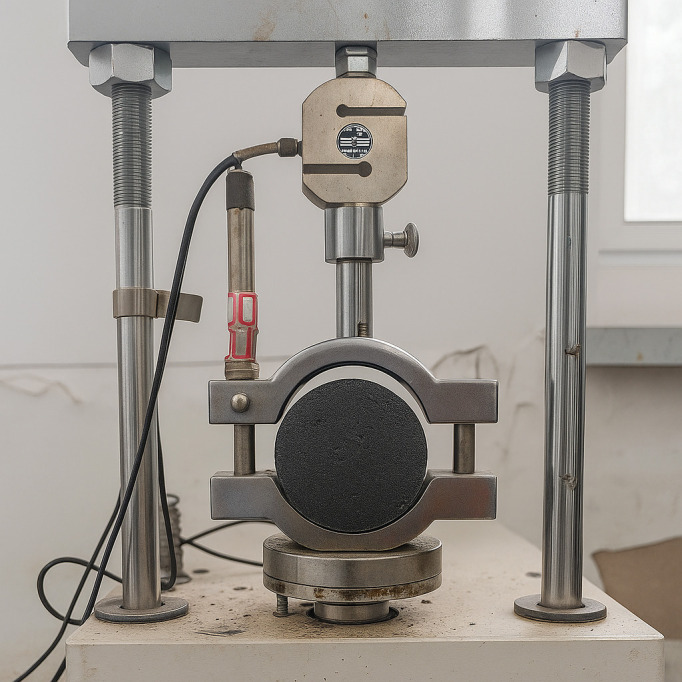
Immersion Marshall test equipment and specimens.

According to standard specifications, Marshall specimens were prepared for base asphalt, SBS-modified asphalt, and ZM-modified asphalt mixtures by applying 75 blows on each face, as shown in Fig 8. The specimens were then divided into two groups: One group was placed in a 60°C water bath for 30–40 minutes for conditioning, and its Marshall stability (MS) was measured.

The other group was immersed in a 60°C water bath for 48 hours, and its stability (MS1) was measured afterward. The residual stability (MS0) was used to evaluate the long-term moisture resistance of the asphalt mixtures under water immersion conditions.


$$MS_{0}=\frac{MS_{1}}{MS}\times 100$$ (5)

(b) Freeze–Thaw Splitting Test

**Fig 9:**
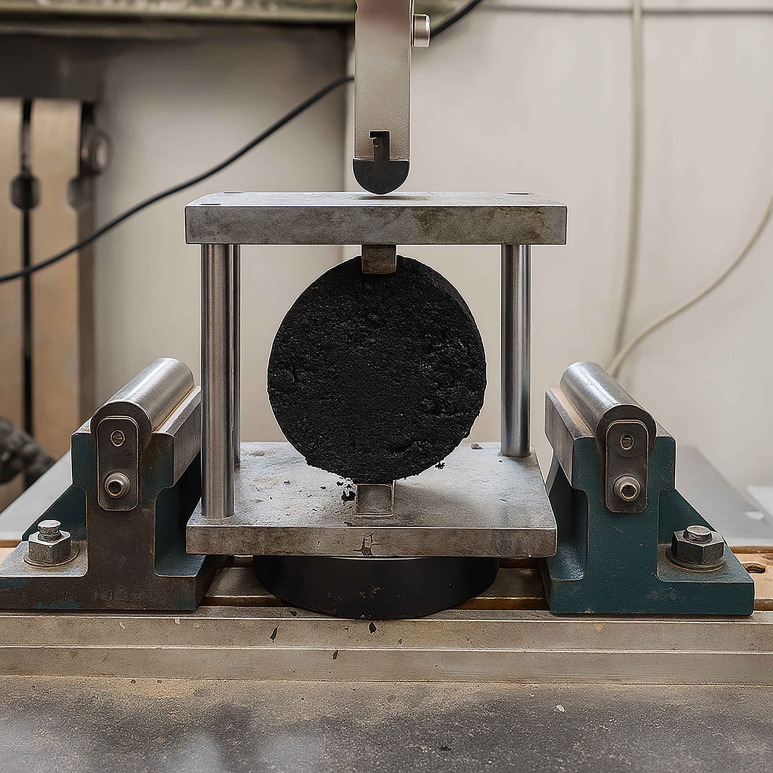
Freeze–thaw splitting test equipment and specimens.


According to the specification, Marshall specimens of base asphalt, SBS-modified asphalt, and ZM-modified asphalt mixtures were prepared by applying 50 blows to each face, as shown in Fig 9. The specimens were divided into two groups: The control group was conditioned in a 25°C water bath for 2 hours, after which the indirect tensile strength was measured. The freeze–thaw group underwent vacuum saturation at 93.7–98.7 kPa for 15 minutes, followed by a 30-minute water soak. The specimens were then sealed in plastic bags with 10 mL of added water and frozen at −18°C for 16 hours. After freezing, they were placed in a 60°C water bath and finally conditioned in a 25°C water bath for 2 hours. The indirect tensile strength of both groups was measured using a universal testing machine. The Tensile Strength Ratio (TSR) was calculated using Equation (6) to evaluate the moisture stability of the mixtures under freeze–thaw conditions.

$$TSR=\frac{R_{2}}{R_{1}}\times 100$$ (6)

In the equation:

*TSR*: Freeze–thaw splitting strength ratio, %.

*R*_*1*_: Average splitting tensile strength of valid specimens without freeze–thaw cycles, MPa.

*R*_*2*_: Average splitting tensile strength of valid specimens after freeze–thaw cycles, MPa.

(5) Fatigue Performance Test
  (a) Semi-circular bending strength test

Before conducting the Semi-circular bending fatigue test, a strength test was performed to determine the appropriate stress ratio for subsequent loading.

Asphalt mixture is highly sensitive to temperature variations, and its strength and fatigue resistance can change significantly with environmental temperature. Studies have shown that fatigue cracking in asphalt pavements typically occurs at the midrange of the pavement’s temperature profile. Based on statistical analysis, the fatigue-equivalent temperature for asphalt pavements in China has been determined to be 15°C [27]. Therefore, the test temperature in this study was set at 15°C.

According to domestic and international standards, a loading rate of 50 mm/min is commonly adopted. Hence, the loading rate for the Semi-circular bending strength test in this study was set to 50 mm/min.

At the beginning of the test, three different types of semi-circular specimens were placed in a temperature-controlled chamber maintained at 15°C for at least four hours. A spacing of 5 cm was maintained between each specimen. This conditioning process continued until the internal and external temperatures of all specimens reached 15 ± 0.5°C. The span between the supports used for testing was 80 mm.

(b) Fatigue Test Plan

The design of the semi-circular bending fatigue test plan in this study includes the following key aspects: environmental temperature, loading control mode, stress ratio (load level), loading frequency, and loading waveform.

(1) Environmental Temperature

Asphalt mixtures are highly sensitive to temperature variations, and their resistance to fatigue failure is significantly influenced by ambient temperature. Therefore, the semi-circular bending fatigue test temperature was set to 15°C.

(2) Loading Control Mode

This study adopted the stress-controlled loading mode, in which a constant stress level is maintained throughout the test until the specimen fails completely.

(3) Stress Ratio (Load Level)

Selecting an appropriate stress ratio is critical in fatigue testing. The stress ratio is defined as the ratio of the peak load applied in a loading cycle to the ultimate load-bearing capacity of the specimen. If the stress ratio is too high, it leads to a shortened fatigue life and premature failure, which does not reflect realistic service conditions. Conversely, an excessively low stress ratio results in prolonged fatigue life and excessively long test durations, which can hinder test efficiency. Therefore, stress ratios of 0.2, 0.3, and 0.4 were selected for the fatigue test in this study.

The semi-circular bending (SCB) fatigue tests were conducted in accordance with JTG E20-2011 (T0739) and AASHTO T321 standards under stress-controlled loading at 15°C. Each stress ratio condition was tested with three specimens, and outlier data were excluded before averaging. The fatigue life results were analyzed using logarithmic regression to establish the fatigue equations and evaluate statistical significance across different mixtures.

(4) Loading Frequency and Waveform

During fatigue testing, the fatigue behavior of asphalt mixtures is significantly influenced by the loading frequency and waveform. Adjusting the loading frequency allows simulation of variations in vehicle speed on road surfaces. Currently, the primary method for determining the loading frequency in laboratory-scale fatigue tests is based on the Vander Poel equation [64]:


t=1/2πf (7)

In the equation:

*t*:loading duration per cycle (s),

*f*:loading frequency (Hz).

In the current experimental study, a loading frequency of 10 Hz is widely adopted. According to Equation (7), the loading duration under a 10 Hz frequency is approximately 0.016 seconds, which corresponds to the contact time between the vehicle and pavement when driving at 60 km/h. Since 60 km/h is a relatively common driving speed in real-world conditions, this setting effectively simulates actual traffic loading scenarios. Therefore, 10 Hz is selected as the loading frequency for this fatigue test.

**Fig 10:**
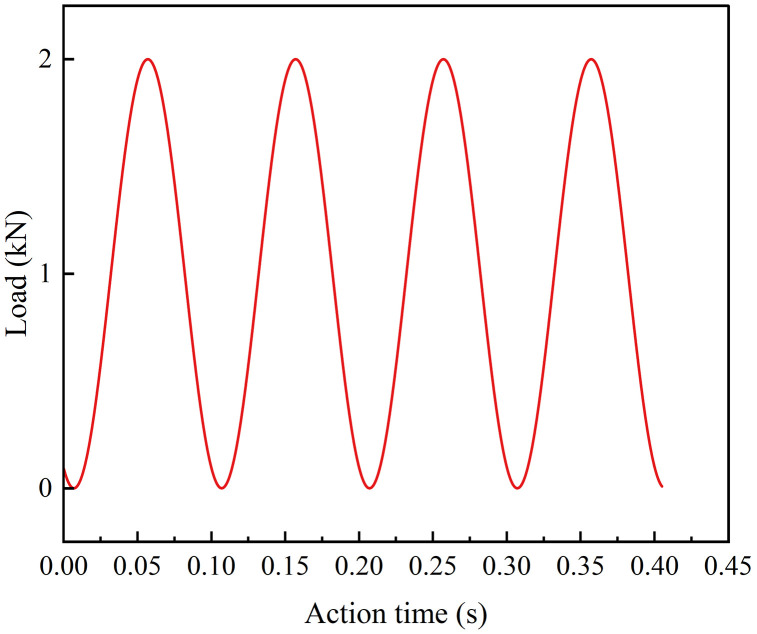
Loading pattern for fatigue test (2 kN load level).

To better reflect the vehicle load patterns experienced by real pavements, a half-sine waveform without rest periods is used. The peak value corresponds to the designated load level, while the valley value is set to a contact force of 0.1 kN. Taking a load level of 2 kN as an example, the loading pattern used in the semicircular bending fatigue test is illustrated in Fig 10.

## 3. Results and discussion

### 3.1. Analysis of orthogonal test results

The dynamic stability of the asphalt mixture was used as the evaluation index in the test. The rutting specimens were prepared using the wheel compaction method with dimensions of 300 mm × 300 mm × 50 mm. The detailed test results are presented in Table 10.

**Table 10:** Results of the orthogonal test.

Test number	*T*(°C)	*t*_1_(s)	*t*_2_(s)	Dynamic Stability (cycles/mm)
1	1 (160)	1 (240)	1 (120)	2719
2	1	2 (180)	2 (180)	2799
3	1	3 (120)	3 (240)	2811
4	2 (170)	2	3	3546
5	2	3	1	3349
6	2	1	2	3147
7	3 (180)	3	2	2949
8	3	1	3	3015
9	3	2	1	3156
Kj1	8329	8881	9224	
Kj2	10042	9501	8895	
Kj3	9120	9109	9372	
${\bar{K}}_{j1}$	2776.33	2960.33	3074.67	
${\bar{K}}_{j2}$	3347.33	3167.00	2965.00	
${\bar{K}}_{j3}$	3040.00	3036.33	3124.00	
Rj	571.00	206.67	159.00	

Kj₁ represents the sum of the test indices at level 1 for the j-th factor column in the table. ${\bar{K}}_{j1}=K_{j1}/3$, $R_{j}=\max\{{\bar{K}}_{j1},{\bar{K}}_{j2},{\bar{K}}_{j3}\}-\min\{{\bar{K}}_{j1},{\bar{K}}_{j2},{\bar{K}}_{j3}\}$

The larger the value of Rj, the greater the influence of the corresponding factor on the test results. Based on the magnitude of the range in the table, the priority ranking of the factors can be determined as follows: mixing temperature > mixing time of modifier and aggregate > total mixing time after addition of asphalt and mineral filler.

Under various factor conditions, the optimal combination of parameters can be identified through comparative analysis of the performance indicators, and suitable processes can be adopted to achieve optimal performance. A higher dynamic stability indicates better test performance under that factor level. Each factor has a corresponding optimal level, and the optimal combination derived from the table is a mixing temperature of 170°C, a dry mixing time of 180 seconds, and a total mixing time of 240 seconds after adding asphalt and mineral filler.

Analysis of experimental data reveals that mixing temperature has a significant effect on dynamic stability. When the temperature is set at 160°C, the dynamic stability is relatively low, likely due to insufficient softening of the modifier at this temperature, which prevents it from functioning effectively. On the other hand, a higher temperature of 180°C may lead to early aging of part of the asphalt mixture. By comparison, a mixing temperature of 170°C yields the best performance. Further research into the modification effect of ZM modifiers shows that it can significantly increase the viscosity of asphalt. Although directly dry-mixing ZM modifiers with aggregates is not identical to modifying the asphalt binder itself, the modifier still exerts a notable influence on the asphalt binder within the mixture.

Adjusting the mixing duration is also crucial. If the mixing time between the modifier and aggregate is insufficient, uniform dispersion of the modifier cannot be achieved. An appropriate duration ensures that the modifier effectively adheres to the aggregate surface during mixing, thereby enhancing its performance once the asphalt is added. Although the mixing duration after adding asphalt and mineral filler is relatively short, it is essential to ensure that the asphalt and filler are evenly coated and thoroughly cover the aggregate, thereby ensuring the finished asphalt pavement achieves the desired in-service performance.

**Fig 11:**
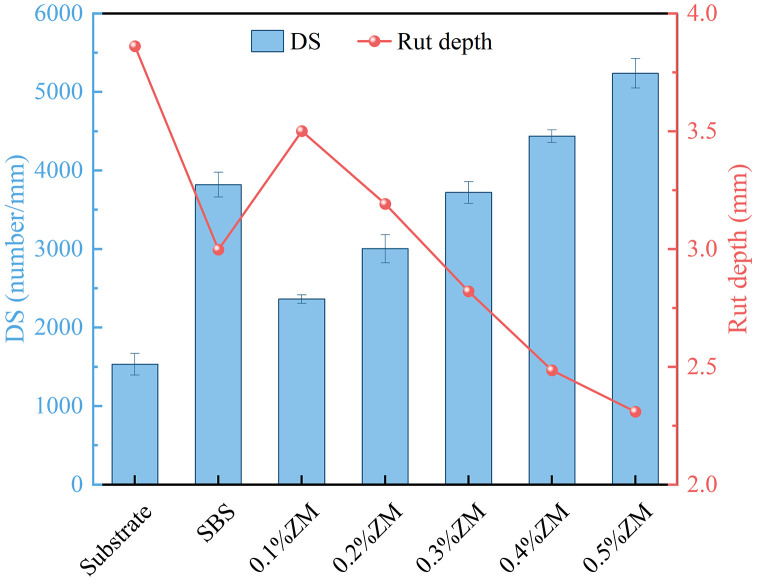
Rutting test results.

The rutting test data are summarized in Table 11, and the corresponding results are illustrated in Fig 11.

**Table 11:** Rutting test results of asphalt mixtures.

Mixture type	Rut depth/mm	Dynamic stability (passes/mm)
Base asphalt mixture	3.81	1530
SBS modified asphalt mixture	3.03	3816
0.1%ZM Modified asphalt mixture	3.51	2361
0.2%ZM Modified asphalt mixture	3.19	3016
0.3%ZM Modified asphalt mixture	2.83	3664
0.4%ZM Modified asphalt mixture	2.49	4432
0.5%ZM Modified asphalt mixture	2.31	5245

The lower the rutting depth and the higher the dynamic stability value of an asphalt mixture, the better its resistance to deformation under high-temperature conditions. As shown in Fig 11, with a 0.1% dosage of ZM modifier, the dynamic stability improves by 54% compared to the base asphalt mixture, and the rutting depth decreases by 9%. This improvement continues with increasing dosage, reaching a dynamic stability of 5245 and a rutting depth of 2.26 mm at a 0.5% ZM content. Additionally, the results indicate that the performance of the mixture with 0.3% ZM modifier is comparable to that of the SBS-modified asphalt mixture. At higher dosages, the high-temperature stability of the ZM-modified mixture surpasses that of the SBS-modified one. This enhancement is attributed to the partial blending of modifier particles with asphalt during high-temperature mixing, which raises the softening point, reduces temperature sensitivity, and improves deformation resistance. Moreover, during mixing and compaction, the ZM particles soften and become viscoelastic due to elevated temperatures, allowing them to fill the voids in the mixture. Upon cooling, the deformed particles harden, resist further deformation, and limit the relative sliding of aggregates, thereby significantly enhancing the mixture’s resistance to rutting compared to base asphalt mixtures.

### 3.2. Analysis of beam bending test results

**Fig 12:**
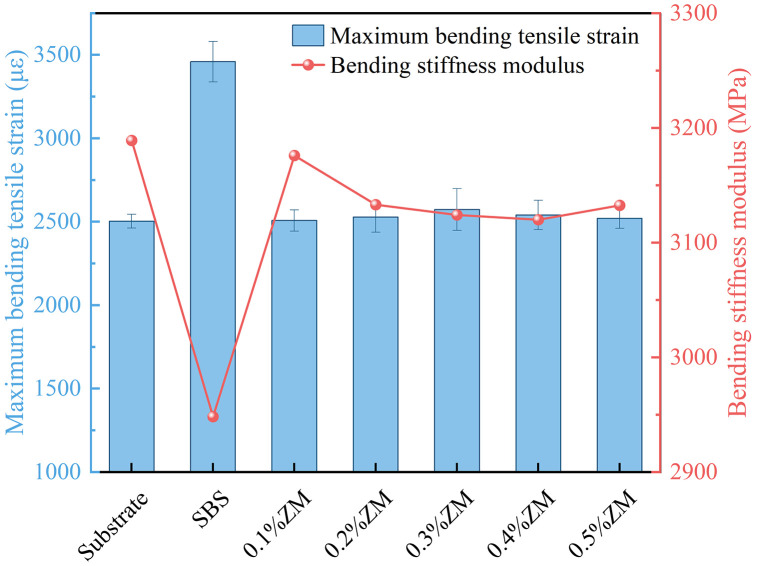
Bending test results of beam specimens.

The experimental results are illustrated in Fig 12.

The maximum bending strain and flexural stiffness modulus at failure were measured in the experiment, both of which are critical indicators for evaluating the low-temperature cracking resistance of asphalt mixtures. Generally, a lower stiffness modulus combined with a higher maximum bending strain implies slower stress accumulation, indicating better low-temperature performance.

As shown in Fig 12, after the incorporation of the ZM modifier, both the maximum flexural strain and the flexural stiffness modulus meet the specification requirements. Compared with the base asphalt mixture, the maximum flexural strain of the ZM-modified mixtures shows a slight increase, while the stiffness modulus demonstrates a modest decrease. These changes indicate that the modifier improves the cracking resistance of the asphalt mixture to some extent. As the dosage increases, the low-temperature flexural strain initially increases slightly, then tends to stabilize when the ZM content reaches 0.3%, suggesting that further improvements in low-temperature performance are limited beyond this dosage.

The ZM additive-composed of thermoplastic polymer and resin-promotes a denser polymer network within the binder mastic and elevates the softening point, which increases stiffness at low temperatures. This stiffening effect explains the slight reduction in maximum bending strain and the stabilization of the flexural stiffness modulus when the dosage exceeds 0.3%. Meanwhile, improved adhesion at the asphalt–aggregate interface helps delay crack initiation; however, once microcracks nucleate, the reduced segmental mobility limits crack blunting, yielding the observed trade-off between enhanced stability and slightly poorer ductility at low temperatures.

### 3.3. Analysis of moisture stability test results

**Fig 13:**
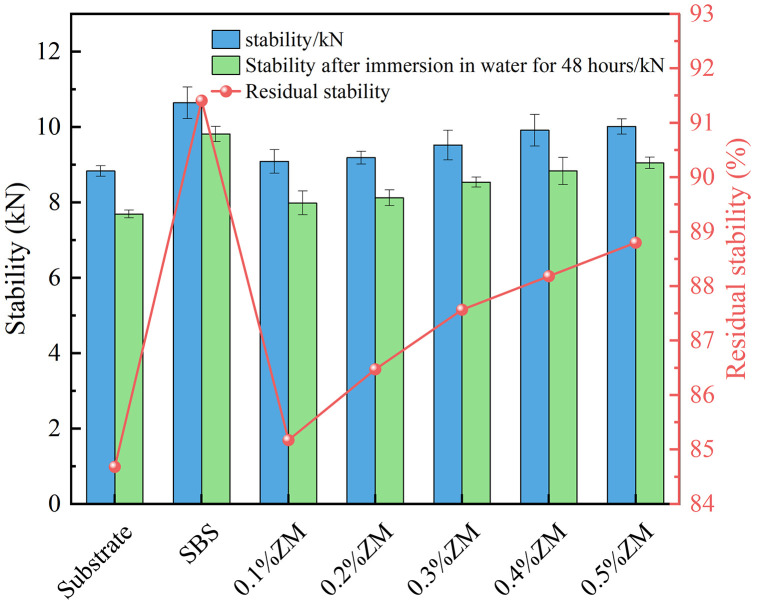
Immersion Marshall test results.

**Fig 14:**
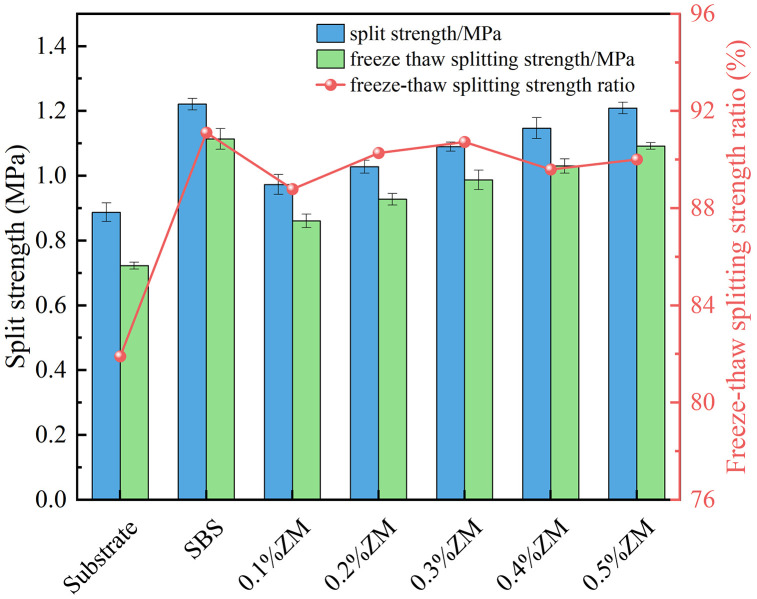
Freeze–thaw splitting test results.

The results of the Immersion Marshall test and the Freeze–Thaw Splitting test are presented in Figs 13 and 14.

In both the immersion Marshall test and the freeze–thaw splitting test, higher values of residual stability and tensile strength ratio (TSR) indicate better moisture resistance of the asphalt mixture. As shown in the Figs, the addition of ZM modifier improves the stability of the modified asphalt as well as the 48-hour immersion stability, leading to an increase in residual stability. When the ZM modifier dosage reaches 0.5%, the residual stability rises from 84% for the base asphalt to approximately 88%. Similarly, the TSR increases from 82% to 90%, an enhancement of 8%. All TSR values for ZM-modified asphalt mixtures exceed 80%, meeting the standard requirements. This demonstrates that the incorporation of ZM modifier enhances the moisture resistance of asphalt mixtures, improving their resistance to water-induced damage.

The stability of asphalt mixtures decreases after 48 hours of water immersion, and the splitting strength is reduced following freeze–thaw cycles compared to the pre-cycle state, indicating that longer water immersion and freeze–thaw durations are negatively correlated with the mixture’s moisture stability. Overall, the moisture stability test results show that the ZM modifier significantly improves the water resistance of asphalt mixtures. This improvement is attributed to the partial fusion of ZM modifier particles with asphalt components during high-temperature mixing, where they enter a viscous flow state, forming a gel-like structure that enhances the adhesion between asphalt and aggregates, thereby improving resistance to moisture damage.

Mechanistic interpretation: moisture damage resistance. The polymer–resin system in ZM enhances mastic cohesion and asphalt–aggregate adhesion, resulting in improved coating uniformity and reduced susceptibility to stripping. The higher softening point and lower drain-off under mixing/compaction conditions improve the continuity of the binder film, thereby limiting moisture ingress and adhesive failure. Consequently, the Immersion Marshall residual stability and the freeze–thaw TSR increase, indicating that ZM primarily strengthens the interface against water-induced debonding.

### 3.4. Strength test results

To ensure the reliability of the test results, three parallel tests were conducted for each type of specimen. In cases where significant deviations were observed, additional parallel tests were performed to supplement the data. The results are presented in Table 12.

**Table 12:** Strength test results of semi-circular specimens.

Specimen type	Failure Load(kN)	Mean failure load(kN)	Standard deviation	Coefficient of variation
Base asphalt mixture	3.654	3.8	0.13	3.3%
	3.821			
	3.905			
0.3% ZM-modified asphalt mixture	4.115	4	0.11	2.7%
	4.033			
	3.894			
0.5% ZM-modified asphalt mixture	4.033	4.1	0.06	1.6%

### 3.5. Fatigue test result analysis

#### 3.5.1. Analysis of fatigue test results.

**Fig 15:**
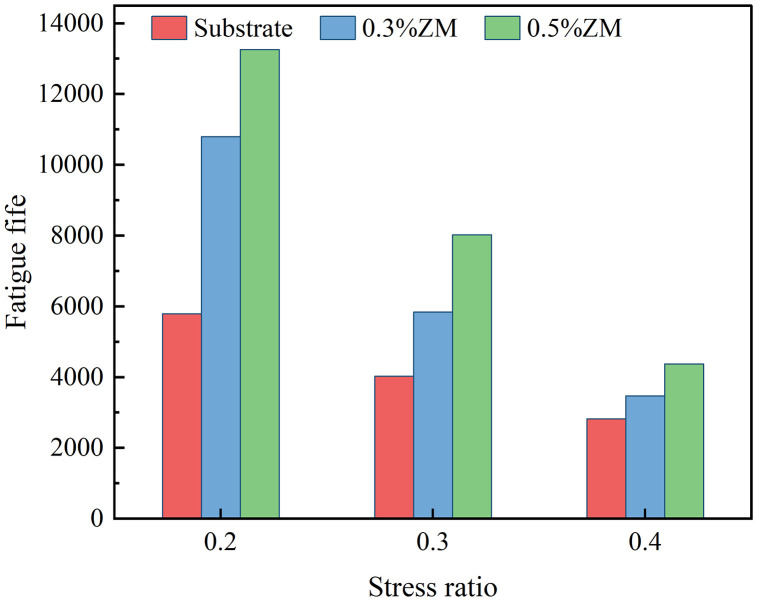
Fatigue life under different stress ratios.

Fatigue tests were conducted on three types of specimens under the predetermined stress ratios, loading frequency, and loading waveform. For each specimen type and stress ratio, three sets of parallel tests were performed. During the testing process, data with high dispersion were excluded, and additional supplementary tests were conducted to ensure accuracy. The final results were obtained by averaging the valid data. The outcomes of the semicircular bending fatigue tests are summarized in Fig 15.

From the analysis of Fig 15, it is evident that the fatigue life of asphalt mixtures decreases with increasing stress ratio. Moreover, under the same stress ratio, the addition of ZM modifier significantly enhances the fatigue life of asphalt mixtures. At a stress ratio of 0.2, the fatigue life of asphalt mixture containing 0.3% ZM modifier increases by 86.1% compared to the base asphalt mixture without the modifier. At a stress ratio of 0.4, the improvement reaches 21.8%. The enhancement in fatigue life becomes more pronounced as the dosage of ZM modifier increases. When the dosage is increased to 0.5%, the fatigue life improves by 128.9% and 56.3% under stress ratios of 0.2 and 0.4, respectively.

#### 3.5.2. Establishment of fatigue equations.

In this fatigue test, semi-circular specimens were subjected to different load levels. To facilitate comparison and analysis of the fatigue test results, the load level–fatigue life data from the table were fitted using equation (5–2), and the results were plotted on a log–log coordinate system.


$$\mathrm{lg}N_{f}=k-n\mathrm{lg}t$$ (8)

In the equation:

*N*_*f*_—Fatigue life (number of cycles);

*t*—Stress ratio;

*n*, *k*—Regression coefficients to be fitted, where n reflects the sensitivity of fatigue life to the stress level.

**Fig 16:**
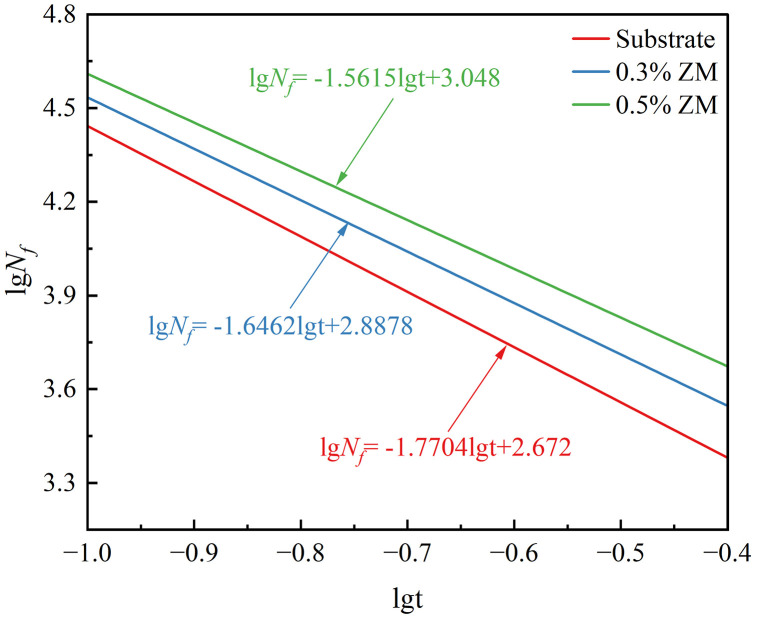
Relationship curve between stress ratio and fatigue life.


By fitting the experimental data in the above table, the following regression results were obtained for the base asphalt mixture, 0.3% ZM modified asphalt mixture, and 0.5% ZM modified asphalt mixture, respectively. The fitted fatigue equations were plotted on the same coordinate system, as shown in Fig 16.

$$\mathrm{lg}N_{f}=-1.7704\mathrm{lg}t+2.672,R^{2}=0.9946$$ (9)

$$\mathrm{lg}N_{f}=-1.6462\mathrm{lg}t+2.8878,R^{2}=0.9972$$ (10)

$$\mathrm{lg}N_{f}=-1.5615\mathrm{lg}t+3.048,R^{2}=0.9957$$ (11)

The above three equations can be transformed to derive the fatigue equations of the three types of semicircular specimens based on the stress ratio:


$$N_{f}=469.89t^{-1.7704}$$ (12)

$$N_{f}=772.32t^{-1.6462}$$ (13)

$$N_{f}=1116.86t^{-1.5615}$$ (14)

Based on the results derived from the fatigue test graphs and fitted equations, the following conclusions can be drawn:

(1) Analyzing the relationship between the fatigue life of semicircular specimens and the stress ratio (load level) reveals a negative correlation—fatigue life decreases as the stress ratio increases. In a log–log coordinate system, this relationship appears as a well-fitted linear curve, indicating a logarithmic linearity between fatigue life and stress ratio. As the ZM modifier content increases, the corresponding curve shifts upward and to the right in the log–log plot, which is reflected in an increased intercept in the fatigue equation. This shift suggests that at the same stress ratio, asphalt mixtures incorporating higher ZM contents exhibit longer fatigue life. Therefore, the addition of ZM significantly enhances the fatigue performance of base asphalt mixtures.
(2) In the fitted fatigue equations, the slope parameter n indicates the sensitivity of fatigue life to the stress ratio. The slope values n for the three types of asphalt mixtures are −1.7704, −1.6462, and −1.5615, respectively. The decreasing absolute values of n suggest that the ZM modifier reduces the sensitivity of fatigue life to changes in stress ratio. This implies that in practical pavement applications, ZM-modified asphalt mixtures may exhibit better durability under higher traffic loads.
(3) Fatigue evolution curves:

During the semicircular bending fatigue test, the linear variable displacement transducer (LVDT) embedded in the loading head automatically records the displacement at the loading point. These data not only capture the number of fatigue load cycles accurately, but also document the full displacement history from the initial loading to the final failure of the specimen. Analyzing the displacement evolution curves provides deeper insights into the progressive damage and state changes of the specimens throughout the semicircular bending fatigue test.

Mechanistic interpretation: fatigue improvement. With ZM, the fitted fatigue equations show a decrease in the absolute slope and an increase in the intercept, implying reduced stress sensitivity and longer life at a given stress ratio. This behavior is consistent with a more elastic mastic response: the polymer network increases instantaneous recovery and reduces the dissipated energy per cycle, delaying microcrack coalescence and propagation. The improved interface adhesion also distributes tensile stresses more uniformly across the skeleton, further extending fatigue life.

**Fig 17:**
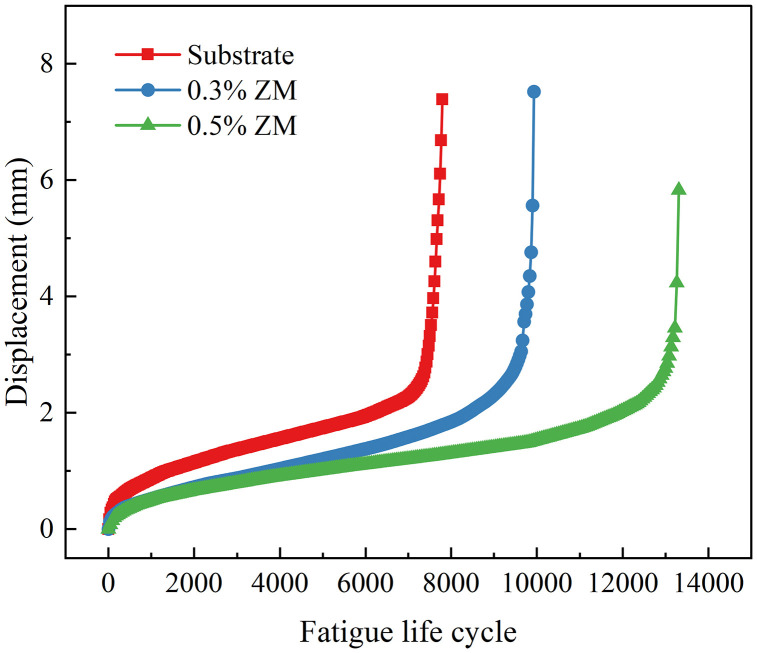
Displacement curve of fatigue Test (stress ratio 0.2).

**Fig 18:**
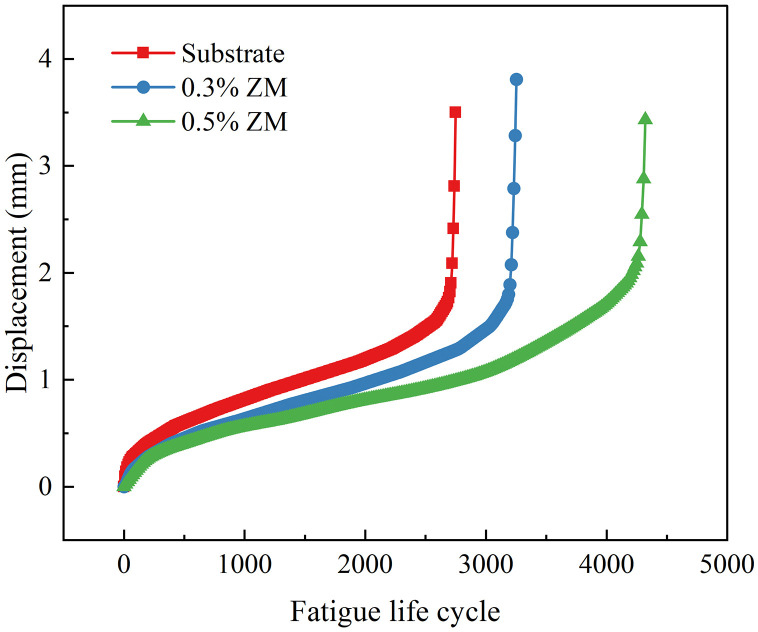
Displacement curve of fatigue test (stress ratio 0.4).

The displacement evolution at the loading point of semi-circular specimens under fatigue testing with stress ratios of 0.2 and 0.4 is depicted in Fig 17 and Fig 18, respectively. These Figs illustrate the development and progression of loading point displacement as a function of loading cycles during the fatigue process.

Throughout the fatigue tests, the displacement at the loading point of each specimen exhibited a distinct three-stage trend as the number of load cycles increased. This trend was characterized by two inflection points on the displacement curve, resulting in an overall reverse S-shaped pattern. In the initial stage, corresponding to the early phase of loading, the displacement increased rapidly with the onset of cyclic loading. This indicated the initiation of plastic deformation and the formation of microcracks. As loading continued, the curve entered a stable growth phase, during which the displacement increased almost linearly. At this stage, crack propagation occurred at a stable rate. This phase constituted the majority of the specimen’s fatigue life. In the final stage, as the applied load persisted and the specimen approached failure, the displacement curve reached its second inflection point. Crack propagation accelerated, and the loading point displacement increased sharply until the specimen ultimately failed due to fatigue.

Among the three types of semi-circular specimens tested at the same stress ratio, those incorporating ZM modifier demonstrated longer fatigue lives, smaller slope values during the second phase of the displacement curve, and larger total displacements at failure. These results indicate that the ZM modifier enhanced the bonding strength between aggregates and asphalt, thereby improving the vertical load-bearing capacity of the specimen. This improvement was manifested by the increased displacement observed at the point of failure.

## 4. Conclusion

(1) The ZM modifier significantly enhances the high-temperature stability of asphalt mixtures. At an optimal dosage of 0.5%, the dynamic stability increases by about 54% and rut depth decreases by nearly 9%, indicating markedly improved rutting resistance under high-temperature conditions.
(2) The incorporation of ZM modifier improves the low-temperature crack resistance of asphalt mixtures. For example, the maximum flexural strain increases by around 5%, while the stiffness modulus decreases slightly, suggesting enhanced resistance to thermal stress–induced cracking.
(3) The ZM additive strengthens asphalt–aggregate interfacial bonding, thereby improving moisture stability. At 0.5% dosage, the retained stability and freeze–thaw splitting strength ratio increase to 88% and 90%, respectively, both meeting specification requirements.
(4) The fatigue performance of asphalt mixtures is significantly improved with ZM addition. Fatigue life is extended by up to 128.9% at low stress ratios, and fatigue sensitivity is reduced, leading to better durability under repeated loading.

While this study provides systematic insights into the effects and mechanisms of the ZM modifier under controlled laboratory conditions, certain limitations remain. The results may vary under field conditions due to differences in traffic loading, environmental temperature, and aging. Future work will focus on long-term field monitoring of ZM-modified pavements, coupled with microscopic and spectroscopic analyses (e.g., SEM, FTIR) to further verify the modification mechanisms and assess durability under real-world service environments.
